# *p*-Methoxycinnamic Acid Diesters Lower Dyslipidemia, Liver Oxidative Stress and Toxicity in High-Fat Diet Fed Mice and Human Peripheral Blood Lymphocytes

**DOI:** 10.3390/nu12010262

**Published:** 2020-01-20

**Authors:** Raquel Teixeira Terceiro Paim, Paula Salmito Alves Rodrigues, José Ytalo Gomes da Silva, Valdir Ferreira de Paula Junior, Bruno Bezerra da Silva, Claísa Andréa Silva De Freitas, Reinaldo Barreto Oriá, Eridan Orlando Pereira Tramontina Florean, Davide Rondina, Maria Izabel Florindo Guedes

**Affiliations:** 1 Biotechnology & Molecular Biology Laboratory, State University of Ceara, Fortaleza 60.714-903, Brazil; paulasalmito@yahoo.com.br (P.S.A.R.); brunoxbezerra@gmail.com (B.B.d.S.); claisa.freitas@uece.br (C.A.S.D.F.); eridan.pereira@uece.br (E.O.P.T.F.); florinfg@uol.com.br (M.I.F.G.); 2 Postgraduate Program in Veterinary Sciences, Faculty of Veterinary Medicine, State University of Ceara, Fortaleza CE 60.714-903, Brazil; biogene.gnr@gmail.com; 3 Laboratory of Tissue healing, Ontogeny and Nutrition, Department of Morphology and Institute of Biomedicine, Federal University of Ceara, Fortaleza 60.430-270, Brazil; oria@ufc.br; 4 Laboratory of Nutrition and Ruminant Production, State University of Ceara, Fortaleza 60.714-903, Brazil; davide@uece.br

**Keywords:** *p*-methoxycinnamic diesters, hyperlipidemia, oxidative stress, lipid metabolism, molecular docking

## Abstract

The pursuit of cholesterol lowering natural products with less side effects is needed for controlling dyslipidemia and reducing the increasing toll of cardiovascular diseases that are associated with morbidity and mortality worldwide. The present study aimed at the examining effects of *p*-methoxycinnamic acid diesters (PCO-C) from carnauba (*Copernicia prunifera*)-derived wax on cytotoxic, genotoxic responses in vitro and on dyslipidemia and liver oxidative stress in vivo, utilizing high-fat diet (HFD) chronically fed Swiss mice. In addition, we evaluated the effect of PCO-C on the expression of key cholesterol metabolism-related genes, as well as the structural interactions between PCO-C and lecithin-cholesterol acyl transferase (LCAT) in silico. Oral treatment with PCO-C was able to reduce total serum cholesterol and low-density lipoprotein (LDL) levels following HFD. In addition, PCO-C reduced excessive weight gain and lipid peroxidation, and increased the gene expression of LCAT following HFD. Furthermore, the high affinity of the studied compound (ΔG: −8.78 Kcal/mol) towards the active sites of mutant LCAT owing to hydrophobic and van der Waals interactions was confirmed using bioinformatics. PCO-C showed no evidence of renal and hepatic toxicity, unlike simvastatin, that elevated aspartate aminotransferase (AST) levels, a marker of liver dysfunction. Finally, PCO-C showed no cytotoxicity or genotoxicity towards human peripheral blood lymphocytes in vitro. Our results suggest that PCO-C exerts hypocholesterolemic effects. The safety of PCO-C in the toxicological tests performed and the reports of its beneficial biological effects render this a promising compound for the development of new cholesterol-lowering therapeutics to control dyslipidemia. More work is needed for further elucidating PCO-C role on lipid metabolism to support future clinical studies.

## 1. Introduction

Cardiovascular diseases (CVDs) prevail as a leading cause of death worldwide, mostly due to an imbalance in plasma lipids levels, especially when associated with other risk factors [1].

High levels of total blood cholesterol (TC), low-density lipoprotein cholesterol (LDL-C), and triglycerides (TG), as well as low levels of high-density lipoprotein cholesterol (HDL-C), characterize dyslipidemia. All together, these factors increase the risk of life-threatening cardiovascular diseases and stroke [2,3].

Among the dietary factors that lead to the development of dyslipidemia, the high consumption of foods enriched in saturated fatty acids are responsible for a significant increase in plasma levels of TC [4]. Therefore, individuals with plasma TC levels above 5 mmol/L or 190 mg/dL are already considered to be hypercholesterolemic, a condition that affects 38.9% of the world population over the age of 25 years [5].

The increasing contribution of medicinal plants and their metabolites to advance cardiovascular disease therapeutics has become even more significant recently. Such metabolites are distinguished by their direct or indirect actions through the activation or inhibition of molecular and cellular targets. Recently, research initiatives are addressing the effects of cinnamic acid derivatives on experimentally hypercholesterolemic animals, because many enzymes that are involved in the production and transport of cholesterol have been shown to be affected by these compounds [6,7,8].

Accumulating evidence has shown that cinnamic acids are able to inhibit HMG-CoA reductase and ACAT enzymes, reduce the high concentration of AST and ALT in serum, lower triglyceride and cholesterol levels, and enhance antioxidants in the erythrocytes and liver of HFD fed animals [9].

The derivatives of cinnamic acids leaves are found in Carnauba (*C. prunifera*) trees. This plant is a palm tree, native to the semi-arid region of the Brazilian Northeast, a part of the riparian forest of this region, where 90 million palm trees thrive in an area of one million sq. Km [10].

Brazil’s main export product, which is derived from carnauba, is the wax powder, boasting more than 16 thousand tons exported in the year 2015 [11]. The wax powder from carnauba is obtained from dried leaves. The powder obtained from carnauba can be used as a substrate for the production of various products in the food, pharmaceutical, cosmetic, automotive, and other industries [12,13,14].

The *p*-methoxycinnamic diesters from Carnauba-derived wax powder, named PCO-C, is a crystalline compound of apolar structure with high antioxidant activity in vitro and with high thermal stability, being obtained from unopened young leaves [15]. PCO-C constitutes 4–7% of the composition of the carnauba wax [15,16].

Previous work from our group revealed important hypolipidemic [17] and hypoglycemic effects [18] of PCO-C. However, its impact on oxidative stress and genes expression involved in lipid metabolism remain unexplored.

This current work addressed in vivo liver oxidative stress, kidney and liver toxicology, other dyslipidemic parameters, and transcriptional levels of key genes that are involved in lipid metabolism. Furthermore, this study explored in vitro cytotoxicity, genotoxicity, and mutagenicity, as well as ligand-enzyme molecular interactions using computational analysis (i.e., molecular docking).

This study aimed to characterize the chemical structure, cytotoxic and genotoxic effects of p-methoxycinnamic acid diesters from Carnauba wax powder (PCO-C) in vitro, as well as to verify hypolipidemic, antioxidant, toxicological, and gene expression effects in a high fat diet fed mouse model. In addition, the affinity energy was evaluated by in silico molecular coupling of PCO-C with LCAT.

## 2. Materials and Methods

### 2.1. Materials and Reagents

The carnauba wax was obtained from Pontes Indústria de Ceras Ltd.a., Fortaleza, CE, Brazil. Cholesterol and cholic acid were purchased from the Sigma-Aldrich Co. (St. Louis, MO, USA). Kits for biochemical analyses of liver (aspartate aminotransferase—AST and alanine aminotransferase—ALT) and kidney (creatinine and urea) function markers were obtained from Bioclin^®^ (Belo Horizonte, MG, Brazil). The reagents ethyl acetate, hexane, and polysorbate 80 (Tween 80) were purchased from the Vetec Quimica Fina Ltd.a—Brazil (Vetec^®^). All of the reagents and solvents were of analytical grade.

### 2.2. Extraction and Characterization of p-Methoxy Cinnamic Acid Diesters from Carnauba Wax Powder

Carnauba wax powder was kindly provided by *Pontes Indústria de Cera Ltd.a*. Separated 100 g of carnauba wax powder were mixed with 300 mL of ethyl acetate and 700 mL of hexane. The mixture was subsequently stirred for 30 min. and then filtered in qualitative filter paper (40 × 40 cm and pore size 26 µm, Prolab^®^). The filtrate material was concentrated in a rotary vacuum evaporator, producing a yellowish compound named PCO-C, which was later analyzed by infrared (IR) spectroscopy and proton nuclear magnetic resonance (^1^H-NMR).

The IR spectra of the compound were assessed while using a VERTEX 70v (Bruker) Fourier transform infrared (FT-IR) spectrophotometer in vacuum. The samples were deposited on the diamond crystal and the spectra were obtained within the absorbance range of 600 to 4000 cm^−1^ with a 4 cm^−1^ resolution in attenuated total reflectance (ATR) mode.

The ^1^H-NMR spectra were recorded at 500.13 MHz with a Bruker Avance DRX-500 Spectrometers^®^ while using a 5-mm dual probe with the tetramethyl silane (TMS) signal as the internal standard and CDCl_3_ as the solvent.

### 2.3. Assessment of In Vitro Cytotoxicity

#### 2.3.1. Isolation of Human Peripheral Blood Lymphocytes (HPBLs)

Heparinised blood was collected from three healthy, non-smoker donors who had not taken any type of drugs/medicines for at least 15 days prior to blood collection and had no history of recent exposure to potentially genotoxic substances (i.e., pesticides, drugs, alcohol, and tobacco) or ionizing radiation (e.g., X-rays) to obtain HPBLs using the method described by Cavalcanti et al. [19]. HPBLs were isolated by the standard density gradient centrifugation method while using Histopaque-1077. The cells were washed, resuspended, and cultured for 24 h in RPMI-1640 medium supplemented with 20% fetal bovine serum, 2 mM glutamine, 100 U/mL penicillin, and 100 µg/mL streptomycin at 37 °C and 5% CO_2_ for use in the PCO-C toxicological tests. Phytohemagglutinin (2%) was added at the beginning of the cell culture period. All of the study reagents for this protocol were from Vitrocell^®^. The Federal University of Ceara approved the protocol regarding healthy human donors (protocol # 281/09). The healthy donors were enrolled either at the Hemocenter of Ceara (HEMOCE) or Clinical Pharmacology Unity of the Federal University of Ceara (UNIFAC/UFC).

#### 2.3.2. Assessment of Cell Viability (MTT Assay)

PCO-C cytotoxicity was assessed in HPBLs from healthy donors while using the 3-(4,5-dimethylthiazol-2-yl)-2,5-diphenyltetrazolium bromide (MTT) (Sigma Aldrich^®^) assay, according to the method that was described by Mosmann [20]. The PCO-C dose range was based on a previous in vitro work from our group [14]. In some of our in vitro studies, we also used lower doses if the compound solubility was viable.

The cells were seeded in clear 96-well plates (1 × 10^6^ cells/well) (TPP^®^). After 24 h, the PCO-C (0.98 to 250 µg/mL), dissolved in a 0.4% Tween-80 solution, was added to the wells and then incubated for 72 h. Doxorubicin (a cytotoxic drug against neoplastic cells) (0.019 to 5 µg/mL) diluted in the same PCO-C vehicle (0.4% Tween-80 solution) was used as a positive control. The medium was replaced by fresh medium (150 µL) containing 0.5 mg/mL MTT. After 3 h, the formazan product was dissolved in 150 mL of DMSO and the absorbance was measured at 595 nm. The assays were conducted twice while using triplicate samples.

#### 2.3.3. Assessment of Haemolytic Alterations

The haemolysis test was performed according to the method that was reported by Costa-Lotufo et al. [21]. A 2% Swiss mouse erythrocyte suspension in 0.85% NaCl and 10 mM CaCl_2_ was plated in 96-well plates. The PCO-C was diluted in 0.4% Tween-80 solution and was tested at concentrations that ranged from 39.06 to 250 µg/mL. Triton X-100 (1%) was used as a positive control. After incubation for 4 h at room temperature (26 ± 2 °C), the samples were centrifuged, and the supernatant was removed and analysed by spectrophotometry at 540 nm for THE Quantification of released haemoglobin.

### 2.4. In Vitro Genotoxicity Assessment Using the Alkaline Comet Assay

The alkaline version (pH > 13) of the comet assay or single cell gel electrophoresis (SCGE) was performed, as described by Singh et al. [22], with slight modifications [23]. Initially, HPBLs (5 × 10^5^ cells/mL) were incubated (37 °C/5% CO_2_) for 24 h with PCO-C (10 to 250 µg/mL; dissolved in 0.4% Tween-80 solution). Doxorubicin (0.3 µg/mL) was used as a positive control and the Tween-80 (vehicle) was used as a negative control. The slides were prepared in duplicates for each concentration of the substance under analysis, and images of 100 cells were randomly selected (50 cells of each duplicate slide) and observed under a fluorescence microscope. The cells were visually assessed and classified into five categories, according to the tail length, as follows: (1) class 0, comets without damage and without tails; (2) class 1, comets with tails shorter than the diameters of the heads (nuclei); (3) class 2, comets with tail lengths 1–2 times the diameters of the heads; (4) class 3, comets with tails more than two times the diameters of the heads; and, (5) class 4, comets without heads. A damage index (DI) value was assigned to each comet according to its category while using the following equation: D = (0 × *n*_0_) + (1 × *n*_1_) + (2 × *n*_2_) + (3 × *n*_3_) + (4 × *n*_4_), where *n* is the number of analysed cells in each class. The damage index ranged from 0 (completely intact: 100 cells × 0) to 400 (with maximum damage: 100 cells × 4).

### 2.5. In Vitro Mutagenicity Assessment Using the Micronucleus Test

The micronucleus test was performed according to the method that was described by Fenech [24]. The HPBLs were treated with PCO-C (10 to 250 µg/mL) for 24 h while using along doxorubicin (0.3 µg/mL) and Tween-80 (0.4%; vehicle) as positive and negative controls, respectively. Subsequently, the cells were washed twice with medium, and cytochalasin-B (3 µg/mL) was added 44 h after starting the culture. At 72 h after adding the drugs, the cultures were centrifuged, and the pellet was resuspended in a 75 mM KCl solution, incubated at 4 °C for 3 min. (mild hypotonic treatment), and fixed using an ice-cold (4 °C) methanol/acetic acid (3:1) fixative solution. This fixation step was repeated twice, and the cells were then resuspended in 1 mL of fixative solution, dripped onto clean and dry slides, stained with 10% Giemsa (pH 6.8) for 6 min., and submitted to microscopic analysis. After fixation and staining by the Leishman method, the mounted slides were analyzed while using light microscopy. Micronuclei (MNs) were typically rounded structures with a diameter of 1/5 to 1/20 of the diameter of the young erythrocytes identified by the bluish color. The micronuclei were identified and counted per 2000 binucleated polychromatic erythrocytes cells (BNCs) with intact cytoplasm were quantified per slide.

### 2.6. Animals and Experimental Diets

Twenty-eight male Swiss mice were used in this study, with six to eight weeks of life and weighing between 25 and 30 g. The mice were obtained from the central vivarium of the Federal University of Ceara and housed at the State University of Ceara vivarium and one-week acclimated.

The animals were kept in polypropylene boxes at 22 ± 2 °C in light/dark cycles (12 h/12 h), receiving a standard diet and water *ad libitum.* The food intake, as well as the body weight of the animals were measured weekly during the experiment. The Ethics Committee on Animal Experimentation of the State University of Ceara approved all of the animal experimental procedures under the protocol number 4558299/2016.

The animals in the normal control (healthy) were fed a standard chow diet (MP-77, Primor, Sao Paulo, Brazil) during the entire study course. This diet was composed of corn, meat meal and soybean meal, wheat bran, sodium chloride (common salt), corn gluten 60, vitamin A, vitamin 12, vitamin D3, vitamin E, vitamin K3, vitamin B2, choline chloride, iron sulfate, copper sulfate, manganese sulfate, zinc oxide, calcium iodate, sodium selenite, BHT (Butylated hydroxytoluene), calcium pantothenate, niacin, and DLmethionine. Table 1 details the composition of the experimental diet.

The high fat diet (HFD) was customized from the standard diet with the addition of butter (10%), cholesterol (1%), and colic acid (0.1%) for the induction of hypercholesterolemia. The addition of butter in the HFD contributed to the increased caloric intake of the experimental mice, as well as cholesterol, because of its enrichment in saturated fat. The HFD used in this study presents 14% lipid against 3% the standard chow diet.

#### 2.6.1. Animal Groups

Swiss mice from HFD, SIMV, and PCO-C groups were fed *ad libitum* a high fat diet for 20 days for the induction of hypercholesterolemia. After this period and confirmation of hypercholesterolemia, the mice continued to receive the diets and they were divided into four groups (*n* = 7), which received the following treatments. They were: (1) by gavage, during 90 days: the standard diet (STD) group was fed with standard chow diet and received water; (2) the high fat diet (HFD) group was fed an enriched fat and cholesterol diet and received water; (3) the SIMV (Simvastatin^®^) group was fed with HFD and received simvastatin (SIMV) at a dose of 20 mg/Kg/day; and, (4) the PCO-C group that was fed with HFD received PCO-C at a dose of 100 mg/Kg/day, by oral gavage. The PCO-C dosage that was utilized in the current study was based on previous reports with beneficial effects in Swiss mice [17,18].

**Figure 1:**
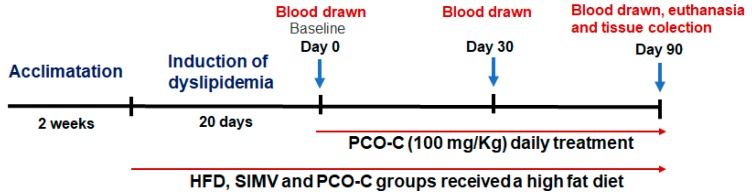
Outline diagram of the in vivo experimental study.

At the end of first and third months of treatment, the animals were fasted for 8 h to analyze blood biochemical parameters. PCO-C was dissolved in a solution with Tween 80 at 3% (*v/v*). Simvastatin was dissolved in water. Euthanasia was performed immediately after the treatment period. The animals were euthanized under ketamine 100 mg/Kg (Kensol^®^) and xylazine 10 mg/Kg (Bayer^®^) anesthesia by cervical dislocation. Figure 1 outlines the in vivo experimental study.

The PCO-C (100 mg/kg) that was used in the present study with mice has equivalent potential in humans if administered at the dose of 8.1 mg/kg, or 486.5 mg/kg/day to a 60 kg adult via normalization to BSA, as guidelines from Food and Drug Administration and Reagan-Shaw, Nihal, and Ahmad, 2007 [25]. This value was estimated from the formula: Human equivalent dose = Animal dose (mg/kg) × Animal Km factor/Human Km factor. The BSA factors of animal Km and Human Km corresponded to values of 3 and 37, respectively [25].

#### 2.6.2. Blood Collection and Biochemical Analyses

The blood samples were harvested using capillary tubes from the retroorbital plexus of the mice, after 30 and 90 days of treatment. After coagulation, the blood was centrifuged 600× *g* for 10 min. The resulting serum was stored at −20 °C until the determination of triglycerides (TG), total cholesterol (TC), high-density lipoprotein (HDL-C), aspartate aminotransferase (AST), alanine aminotransferase, (ALT), glucose, creatinine, and urea. Biochemical analyses were carried out while using Metrolab 23,300 equipment. The Friedewald, Levy and Fredrickson [26] method was used to calculate the value of low-density lipoproteins (LDL-C), and the LDL-C levels were obtained according to the following formula: LDL-C = (CT-HDL-C) − (TGL/5).

### 2.7. Determination of Liver Lipid Peroxidation (MDA Assay)

The liver samples were cut into small fragments and snap frozen at −80 °C until analysis. The liver lipid peroxidation was determined by estimation of malondialdehyde (MDA) activity while using the thiobarbituric acid reactive substances (TBARS) test. The liver tissue was macerated in phosphate buffer solution to prepare a 10% homogenate. A sample of 250 μL of the homogenate was incubated in a water bath at 37 °C for 60 min. After incubation, 400 μL of 35% perchloric acid and the samples were centrifuged at 3000× *g* for 10 min. at 4 °C. 550 μL of the supernatant was added to 200 μL of 0.8% thiobarbituric acid, boiled at 95 ± 1 °C for 30 min. in a water bath and then immediately cooled. Subsequently, the absorbance of this mixture was measured at 532 nm. The standard curve was prepared while using 1,1,3,3-tetra methoxy propanol. The results were expressed in nmoles of MDA per mg of tissue protein [27].

### 2.8. Extraction of Total Hepatic RNA and Complementary DNA Synthesis (cDNA)

After dissection, the liver fragments were immediately stored in RNAlater (Sigma Aldrich, St. Louis, MO, USA) for 24 h and the samples were then transferred and frozen in −80 °C freezer until analyzed. Total hepatic RNA from each animal was obtained while using a specific kit (RNeasy Mini Kit, Qiagen, Hilden, Germany), in compliance with the manufacturer’s instructions. Volume equivalent to 1 μg of total RNA was treated with DNase I (Life Technologies, Carlsbad, CA, USA) and subsequently used for cDNA synthesis while using M-MuLV reverse transcriptase (New England Biolabs Inc., Ipswich, MA, USA).

#### Real Time Quantitative Polymerase Chain Reaction (RTqPCR) Analysis of Hepatic (i.e., Lipid Metabolism Markers)

For the relative quantification of the mRNA of ApoA1, LCAT, HMGCR, LXRα, HPRT, and B2M validated primers were used according to RTPrimerDB database [28] with SYBR Green Real-Time PCR Master Mix reagent (Life Technologies), with each reaction being performed in triplicate (Table 2). The B2M and HPRT were used to normalize the qRT-PCR gene expression.

### 2.9. In Silico Analysis of the Properties of Ligand

Cholesterol, (Id: 5997) and dithiobis-nitrobenzoic acid (DTNB) (Id: 6254), structures were obtained from the PubChem database [29] and the *p*-methoxycinnamic acid diester compound was drawn while using ChemSketch [30]. These structures were submitted to the MarvinSk [31] program for topology analysis, such as: valence check, charge analysis, isomers (resonance), and geometric analysis. Finally, energy minimization was performed by MMFF94 [32] generating a refined model.

#### 2.9.1. Computational Analysis of the Enzyme Lecithin Cholesterol Acyltransferase (LCAT)

The three-dimensional structure of natural normal LCAT (nLCAT) (PDB entry: 4 × 96, chain A) [33] and mutant LCAT (mLCAT) (PDB entry: 4XWG, chain A) [34] with the C31Y mutation was acquired from the RCSB Protein Data Bank (PDB) database [35]. These structures were edited for the removal of water molecules by the VMD Molecular Graphics program [36]. Finally, amino acid characterization of the catalytic pocket of the protein was completed by Accelrys Discovery Studio Visualizer 4.5 software (Dassault Systemes, BIOVIA Corp., San Diego, CA, USA).

#### 2.9.2. Molecular Docking Study

In molecular docking, the AutoDockTools 1.5.6 algorithm [37] was used to add Kollman’s charges on the molecules of Dithiobis-nitrobenzoic acid (DTNB), p-methoxycinnamic acid diester, and to evaluate the freedom of torsion angles by the “tree” command of torsion.

The nLCAT and mLCAT enzymes were optimized by the addition of polar hydrogen and through the calculation of Gasteiger loads. Subsequently, the catalytic pocket of the enzymes was defined with the amino acids of the nLCAT catalytic triad (Ser181, Asp345, and His377), which are located below the lid segment (residues 226–234) that is involved in the protection of the active site [33,34,38].

The catalytic region of the mLCAT enzyme was delimited with a grid cube having an angstrom spacing of 0.375 A and grid center coordinates x: −16.253, y: 33.615, z: −30.849, and the nLCAT enzyme with a Midpoint = (386.0, 17.1, −89.9). These data were saved in an extension file (i.e., gpf).

Finally, the AutoDock 4.2 program developed an evaluation of complementarity (drug-protein). In this way, the binder took a flexible form, having greater freedom of twist with the following parameters: Genetic Algorithm, Simulated Annealing, Local Research Set docking, and Lizard genetic algorithm (LGA) [37], with the benchmark of 50 execution.

#### 2.9.3. Evaluation of the Complex (Drug-Protein)

We selected the best poses of the molecules within the active site of the protein with the lowest binding energy found in Kcal/mol (score = −ΔG) after the benchmark of the program [39]. These data were used to calculate the inhibition constants (Ki) by the equation (ΔG = RT ln Ki), where R = universal gas constant (1987 Kcal/mol) and T = Kelvin system global temperature (15 K). The type of force involved in the complex (drug-protein) and the participant residues were observed by the Accelrys Discovery Studio Visualizer 4.5 [40], while the root mean square deviation analysis (RMSD) was completed while using PyMOL 2.0 (Schrodinger, LLC, New York, NY, USA).

### 2.10. Statistical Analysis

Statistical analyses were performed while using Graphpad Prism software version 7.0 (Intuitive Software for Science, San Diego, CA, USA). The half maximal inhibitory concentration (IC_50_) value of the MTT assay was assessed by non-linear regression. The data from the in vitro and in vivo alkaline comet assays and micronucleus tests were expressed as the mean ± standard deviation (STD) and then compared by analysis of variance (ANOVA), followed by the Student-Newman-Keuls test (*p* < 0.05). The significance of differences among animals from multiple experimental groups (means ± standard error) was assessed while using ANOVA, followed by the *Tukey* test. A value of *p* < 0.05 was considered to be significant.

## 3. Results

### 3.1. Identification of p-Methoxycinnamic Acid Diesters Extracted from Carnauba Wax Powder

The results from the infrared (IR) spectroscopic analyses and ^1^H-NMR enabled the identification of PCO-C as 4-methoxycinnamic acid diester (Table 3), according to the study that was reported by Vandenburg and Wilder [41].

**Figure 2:**
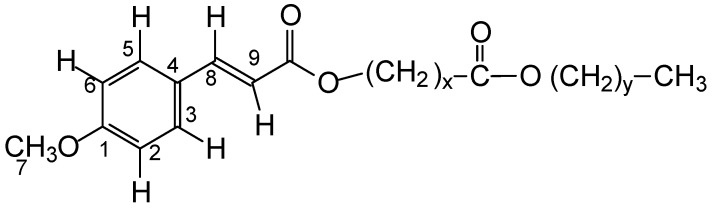
Structural representation of 4-methoxycinnamic acid diester (PCO-C).

The analysis of PCO-C and infrared absorption spectroscopy identified the presence of absorption bands characteristic of ester (1738, 1717, and 1169 cm^−1^), unsaturated (163 and, 930 cm^−1^), p-substituted aromatic (830 cm^−1^), and p-methoxy aromatic (1020 cm^−1^) functional groups. The ^1^H-NMR spectrum and its expansion confirmed that PCO-C is a 4-methoxycinnamic acid ester, according to the spectroscopic data and the structure (Figure 2 and Table 3).

### 3.2. PCO-C Effects on Cell Viability (MTT Assay), Haemolytic Alterations, In Vitro Cytotoxicity, Genotoxicity and Mutagenicity

The results from the PCO-C treatment MTT assay showed no cytotoxicity towards HPBLs at the tested concentrations (IC_50_ > 250 µg/mL).

PCO-C caused no haemolysis of mouse erythrocytes, even at the highest concentration (2500 µg/mL) used.

**Figure 3:**
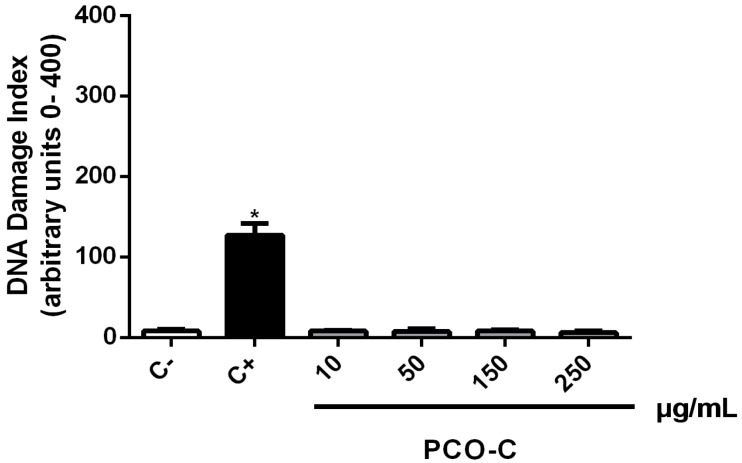
DNA damage index of human peripheral blood lymphocytes treated with PCO-C (10–250 µg/mL) assessed using the alkaline comet assay after 24 h of exposure. For dilution of the test substance, 0.4% Tween-80 was used. Doxorubicin (0.3 µg/mL) and 0.4% Tween-80 were used as the negative and positive controls, respectively. Bars represent the mean ± standard deviation of three independent experiments. * *p* < 0.05; vs. control (0.4%Tween-80) according to ANOVA, followed by *Tukey* test.

Furthermore, PCO-C did not show genotoxic activity against HPBLs with the tested concentrations (from 10 to 250 µg/mL), as shown in Figure 3. PCO-C caused no significant DNA damage (*p* < 0.05) when compared to the negative control (DI = 8.00 ± 2.08), even at the highest concentration (DI = 6.00 ± 2.08). Doxorubicin, which is a well-known anti-cancer cytotoxic drug, was used as a positive control, and it showed expected marked genotoxicity (DI = 127.00 ± 14.73).

PCO-C, even at its highest concentration (250 µg/mL), induced the formation of only 0.33 ± 0.03 micronuclei/2000 binucleated HPBLs, based on micronucleus test (Table 4). Therefore, PCO-C did not induce significant (*p* < 0.05) formation of micronuclei (MN) as compared to the negative control (0.66 ± 0.33 micronuclei/2000 binucleated HPBLs). The positive control, doxorubicin, significantly (*p* < 0.05) induced the formation of micronuclei with 25.66 ± 3.52 micronuclei/2000 binucleated HPBLs. Therefore, PCO-C showed no mutagenic effect in HPBLs with the tested concentrations.

### 3.3. Effect of PCO-C on Blood Lipids from Hypercholesterolemic Mice and Controls

The hyperlipidemic diet was adapted from a previous model that was described by Guedes et al. [15]. This diet has been found to increase murine serum cholesterol levels.

The HFD was effective in increasing serum cholesterol and LDL-C levels. However, this diet did not induce increased triglyceride levels and glycaemia (Table 5).

After 30 days of treatment, PCO-C (100 mg/Kg/day) significantly reduced the TC levels (−18.84%; *p* < 0.05), as compared to the untreated HFD group, as follows: 243.1 ± 7.22 mg/dL (HFD group) and 197.3 ± 6.06 mg/dL (PCO-C group). These results were very similar to those from the group that received simvastatin (−17.16%; 201.4 ± 3.95 mg/dL), which suggests that PCO-C had similar effectiveness in reducing TC levels when compared to the standard drug. Moreover, after 90 days of treatment, a same reduction trend (*p* < 0.05) with PCO-C-treated mice (−19.18%; 189.7 ± 4.82 mg/dL) and simvastatin (−23.44%; 179.7 ± 8.36 mg/dL) was found, when compared to the HFD group (234.7 ± 16.18 mg/dL). The serum TC levels were raised as compared to baseline along time in the standard chow diet and the HFD. However, this effect was not observed in the simvastatin and POC-C groups. Interestingly, PCO-C group showed a significant reduction in cholesterol levels 90 days after treatment compared to the baseline (listed in Table 5).

As for serum LDL-C levels, there was an effective reduction of this parameter after 30 days of treatment, only after PCO-C treatment (−26.7%, 75.94 mg/dL) when compared to the HFD group. Within 90 days, PCO-C and simvastatin-treated mice showed a significant reduction in the LDL-C serum levels. When we examined intra-group changes in serum LDL-C over time, we found that only the HFD group showed significantly increased LDL-C levels (only 90 days after) when compared with the baseline (Table 5).

Interestingly, chronic feeding with the HFD reduced triacylglycerol levels throughout the treatment. In addition, there was a reduction of this parameter after PCO-C and simvastatin treatments, as compared to the HFD group. The serum triacylglycerol levels were raised when compared to baseline along time in the standard chow diet and the HFD (30 and 90 days after), however this effect was not seen with simvastatin and the POC-C group (Table 5).

The simvastatin and PCO-C groups, after 90 days of treatment, had an expressive increase in the HDL-C serum levels. The observed values (mg/dL) were: 84.71 ± 6.40 (+49.91%) and 75.14 ± 10.13 (+43.53%), respectively, as compared to the HFD group (42.43 ± 14.00). Only in the HFD group were HDL levels were found significantly increased over time when compared to the baseline on both days 30 and 90 afterwards.

No changes with PCO-C and simvastatin treatments were seen with the glucose levels when compared to the standard chow diet. However, glycemia was significantly higher in the PCO-C group as compared to the baseline albeit not reaching murine diabetic levels (Table 5).

### 3.4. Effect of PCO-C on Body Weight and Food Intake from Hypercholesterolemic Mice and Controls

The chronically given high-fat diet was able to induce a significant increase (*p* < 0.05) in mice body weight (112.2%), which led to obesity up to the end of the study. However, simvastatin and PCO-C treatment lowered weight gain. The observed values were 42.87 g (−16.7%) and 47.46 g (−7.8%) for the PCO-C and simvastatin, respectively, as compared to the HFD group (51.47 g). Thus, the PCO-C-treated mice gained weight similarly to the simvastatin group following HFD. Moreover, PCO-C was effective in reducing the food intake of the high fat diet, which might account for the lower weight gain of these animals, as compared to the other groups in this study (Table 6).

### 3.5. Effect of PCO-C on Liver and Kidney Function Markers and Liver Lipid Peroxidation (MDA Assay)

Hepatic (AST and ALT) and kidney (urea and creatinine) functions were evaluated, as well as the relative weight of these organs were examined at the end of the experiment, to evaluate the systemic toxicity of PCO-C.

Table 7 shows no significant differences in hepatic and kidney parameters following HFD chronic feeding in untreated and PCO-C-treated mice. Conversely, animals that were treated with the standard cholesterol lowering drug (simvastatin) showed significant hepatic and kidney alterations (*p* < 0.05), with AST elevation and creatinine reduction.

It is worth noting that untreated challenged mice (HFD group) showed significant relative liver weight gain (RLW) (*p* < 0.05), whereas simvastatin and PCO-C-treated HFD-challenged mice did not show significant differences in the liver weight (Table 7).

We evaluated malondialdehyde (MDA) levels, as a marker of hepatic oxidative stress, in order to assess liver peroxidation activity.

As expected, hepatic homogenates of the untreated challenged animals (HFD group) showed high liver lipid peroxidation depicted by high MDA levels (0.39 nmol/g). On the other hand, PCO-C treatment was found to be protective against liver oxidative stress, with significantly lower (*p* < 0.05) MDA levels (0.26 nmol/g), as compared to the HFD group (Table 7).

### 3.6. Gene Expression of Key Lipid Metabolism Markers in Liver Tissue by RTqPCR

**Figure 4:**
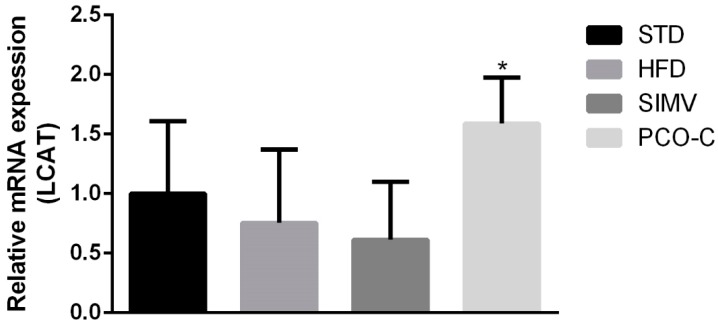
Effect of PCO-C on the transcription levels of liver LCAT (lecithin-cholesterol acyl transferase) mRNA. STD = standard chow diet; HFD = high fat diet; SIMV = simvastatin (20 mg/Kg/day); PCO-C = p-methoxycinnamic acid diesters (100 mg/Kg/day). Values are expressed as mean ± SEM of 7 mice per group. * *p* < 0.05 versus HFD group. Relative gene expression levels were then calculated as 2^−delta delta CT^.

Liver lecithin-cholesterol acyl transferase (LCAT) gene expression showed a slight tendency to change, albeit not significantly, between the experimental groups and in controls that were fed a standard chow diet. Nonetheless, significantly different mRNA levels were observed, with a mean expression ratio PCO-C: HFD of approximately 2.05 (Figure 4).

No changes were observed in the other measured liver markers (APOA-I, LXRα, and HMGCR mRNA levels) in the current study.

### 3.7. Molecular Re-Docking and Docking

**Figure 5:**
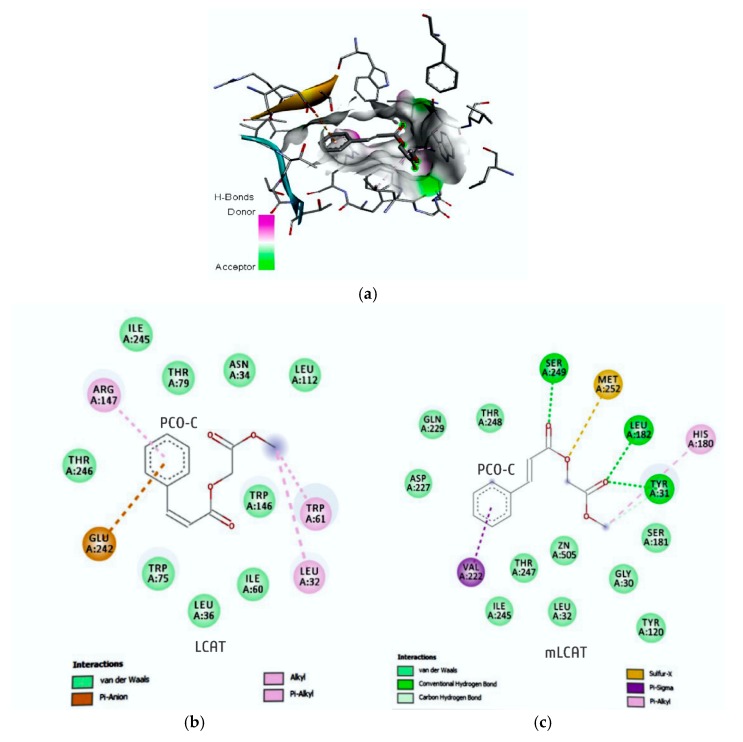
Three-dimensional (3D) (**a**) and two-dimensional (2D) (**b**) arrangement of residues involved in the interaction of PCO-C with nLCAT (presence of Cys31), interaction with mLCAT (**c**) (presence of Tyr31). The residues were observed by the Accelrys visualizer software, version 4.5.

The p-methoxycinnamic acid diester depicted interaction energy of −7.04 Kcal/mol and ki: 6.88 uM (Table 8), with 13 catalytic region residues (nLCAT) (Ile245; Thr79; Asn34; Leu112; Trp146; Trp61; Leu32; Ile60; Leu36; Trp75; Glu242; Thr246; and, Arg147) (Figure 5).

The interaction of the *p*-methoxycinnamic acid diester with the mLCAT was found with a lower interaction energy −8.78 Kcal/mol and ki: 365.47 nM (Table 8), with 11 amino acids (Val222; Ile245; Thr247; Gly30; Leu32; Tyr120; Ser181; Tyr31; His180; Met252; Thr248; Gln229; and, Asp227) interacting with the hydrophobic, van der Waals, and hydrogen bonds of Ser249 a 4.07 angstrom (Å) with O15; Leu182, 3.82 Å-O13 of the compound. A mutation in the residue Cys31-Tyr31 contributed to the interaction of hydrogen with a 4.83 Å-O13 of the compound. In addition, the methionine (Met) residue is able to establish a sulfur bridge with O14 a 4.90 Å (Table 8 and Figure 5).

**Figure 6:**
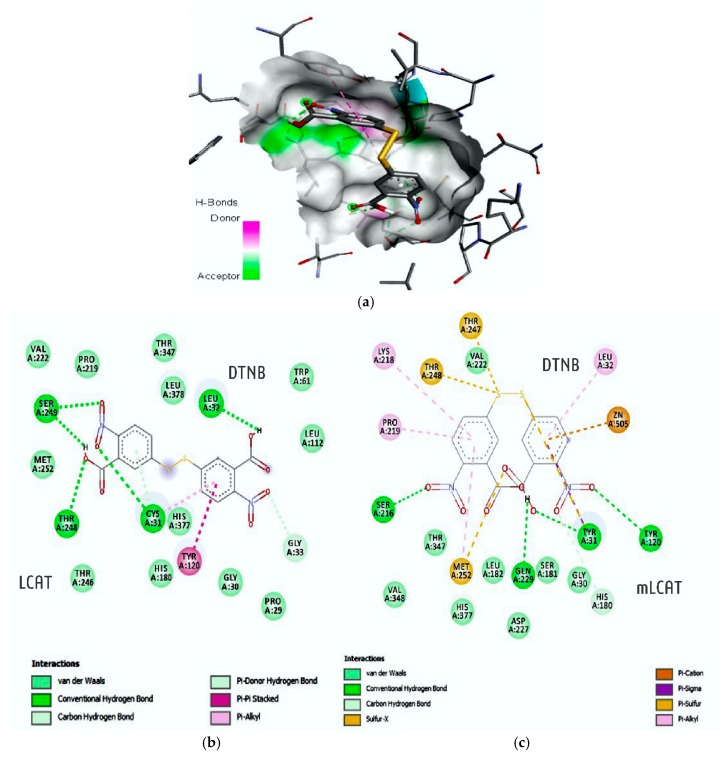
3D (**a**) e 2D (**b**) arrangement of residues involved in the interaction of DTNB with nLCAT (B) (presence of Cys31), interaction with mLCAT (**c**) (presence of Tyr31). Residues were observed by the Accelrys visualizer software, version 4.5.

DTNB exhibited greater complementarity with the nLCAT enzyme with less perturbation to the system, with an interaction energy of −11.44 Kcal/mol e ki: 4.13 nM with mLCAT and −10.03 Kcal/mol, ki 44.64 nM with nLCAT (Table 1). DTNB established favorable interactions with nLCAT, owing to van der Waals and hydrophobic (Gly30; Pro29; Tyr120; Leu378; Thr347; Pro219; Val222; Met252; Thr246; His180; Leu112; Trp61) and hydrogen interactions (Cys31, 5.19 Å-O23; Leu32, 4.80 Å-H27; Thr248, 3.97 Å-O22; Ser249, 2.56 Å-H28, O24) (Figure 6).

However, the simulation to DTNB emerging in the catalytic pocket of mLCAT characterized 11 residues (His180; Gly30; Ser181; Asp227; Leu182; Val348; Thr347; Val222; His377) that partiipate in hydrophobic interactions and van der Waals, (Tyr31, 4.32 Å-O22; Tyr120, 5.93 Å-O23; Gln229, 3.98 Å-H27; Ser216, 3.96 Å-O18), along with the interaction of hydrogen, and sulfur bridge with the repeatable (Met252, 5.33 Å; Thr247, 5.96 Å; Thr248, 5.05 Å) (Figure 6).

DTNB exhibited greater complementarity with the nLCAT enzyme with less perturbation to the system, with an interaction energy of −11.44 Kcal/mol e ki: 4.13 nM with mLCAT e −10.03 Kcal/mol, ki 44.64 nM with nLCAT (Table 1). DTNB established favorable interactions with nLCAT, owing to van.

## 4. Discussion

The diester of *p*-methoxycinnamic acid (named PCO-C) corresponds to only a small fraction of carnauba, a native palm tree that is found in northeastern, Brazil. Our group has successfully studied the pharmacological effects of PCO-C in in vivo and in vitro [14,17,18]. However, our current work brings novelty in highlighting its benefits on liver oxidative stress, hepatic and renal toxicity, and the expression of key genes involved in lipid metabolism. In addition, we did not find any PCO-C related cytotoxic and genotoxic effects to HPBLs. Importantly, cinnamic acid and several of its esters have been reported with no cytotoxicity towards healthy human cells, but with selective cytotoxicity towards malignant cells [42,43]. According to Maistro et al. [44], cinnamic, ferulic, and caffeic acids show no significant cytotoxic effects (using MTT assays) with concentrations that range from 1 to 1500 µM in drug metabolizing hepatoma tissue cells of rat origin. Carvalho et al. [45] synthesized five novel cinnamic N473 acylhydrazone derivatives and found low toxicity (IC50 values ranging from 281.3 ± 14.8 to 2413.0 ± 100 µM) towards murine macrophages, as well as excellent trypanocidal activity (against *Trypanosoma cruzi*).

Our finding that PCO-C shows no haemolytic effect supports a previous study from Jiang and colleagues, who reported the anti-haemolytic activity (in human red blood cells) of cinnamic acid derivatives and their corresponding cinnamaldehydes, according to the following order: o-coumaraldehyde > p-coumaraldehyde ≈ caffeic aldehyde > caffeic acid ≈ o-coumaric acid > p-coumaric acid > vitamin C. This result might be explained by the higher lipophilicity of cinnamaldehydes, which more easily adhere to poly-unsaturated fatty acids in erythrocyte membranes. These authors noted that the degree of lipophilicity might be proportional to their anti-haemolytic activity [46].

In support to our data, Cinkilic et al. [47] show that cinnamic acid protects against radiation-induced genomic instability (1 or 2 Gy) in normal human blood lymphocytes. Nonetheless, it has been recognized that carnauba wax did not show mutagenic effects on *S. cerevisiae* and several *S. typhimurium* strains [48].

In our study, Swiss-fed HFD mice showed elevated levels of LDL-C, as well as greater weight gain and reduction in HDL-C levels when compared with the controls. Other studies using similar HFD also found a significant increase in serum levels of total cholesterol and LDL-C [49,50].

Other compounds with a similar chemical structure to PCO-C, such as the gamma-oryzanol (which is extracted from rice bran oil) and policosanol (present in sugar cane wax), show significant benefits in the reduction of serum lipid levels in Wistar rats and in humans [51].

In the present study, HFD chronic feeding induced a significant increase in total weight gain of Swiss mice. Food intake and body weight are related to the metabolism of macronutrients and they can be used as sensitive markers of general health status [52]. PCO-C treatment was able to significantly reduce total weight gain, as well as food intake, closely resembling those that are induced by simvastatin. We speculate that this weight factor of PCO-C might be due to an anti-adipogenic effect, however with altered leptin response, leading to greater satiety [53]. More studies are warranted to elucidate potential mechanisms to control the food intake and weight gain effect induced by PCO-C.

Untreated mice fed an HFD showed high liver lipid peroxidation. The LDL-C particles can be readily oxidized into small, dense molecules, whose ability to cause cell damage and promote endothelial dysfunction is very significant [54]. PCO-C treatment could reduce lipid peroxidation, with MDA levels close to standard diet controls. Recovery from liver injury usually correlate with antioxidant ability to prevent lipid peroxidation [55]. Our findings support an early study from our group showing marked antioxidant activity of PCO-C in vitro. Furthermore, PCO-C (250 μg/mL) was also able to inhibit the production of reactive oxygen species (ROS) in human peripheral blood lymphocytes [14]. Altogether, these results suggest a relevant electron donation capacity of PCO-C.

It is an interesting finding of this study that cholesterols levels, even under standard chow diet, were increased over time. One early study did show that cholesterol levels may increase with age in different mouse inbred strains [56], even under a standard chow diet. Interestingly, LDL-C levels were not so affected by age. That change is still elusive and it requires further investigation. As expected, the high-fat diet-challenged mice were the ones with significantly higher cholesterol and LDL-C levels as opposed to the STD controls.

Untreated mice that were fed with HFD showed high liver lipid peroxidation, on the other hand PCO-C treatment could reduce lipid peroxidation, with MDA levels that are close to standard diet controls. Our findings support an early study from our group showing a marked antioxidant activity of PCO-C in vitro by the FRAP, ABTS methods, and stimulated gastrointestinal digestion. PCO-C (250 μg/mL) was also able to inhibit the production of reactive oxygen species (ROS) in human peripheral blood lymphocytes. Altogether, these results suggest a relevant electron donation capacity of PCO-C [14].

The experimental high fat diet used chronically on Swiss mice also induced increased liver weight, which suggests fat accumulation (liver steatosis). This effect was not observed with POC-treatment. In addition, urea and creatinine (kidney function) and AST and ALT (liver function) markers were affected by simvastatin, but not with PCO-C. Simvastatin increased AST levels in 2.7 times, as compared to the group that received only standard diet, indicating a possible liver injury.

Liver ALT and AST enzymes are transaminases that are often altered by the chronic use of statins, causing a condition that is known as “transaminitis”, being characterized by liver enzyme abnormalities in the absence of proven hepatotoxicity, so it is not used as a reliable indicator of liver injury [57]. Dujovne [58] pointed out that, in the process of lipid reduction or in situations, such as fatty liver, there is extravasation of liver enzymes by changes in hepatocellular membranes that are caused by the use of statins.

In addition, statins outcome on increasing creatinine levels might be due to its muscle effect, since animals that received simvastatin showed a significant reduction in creatinine levels [59]. Reduced levels of creatinine biosynthesis, by the reduction of GATM (enzyme that is involved in creatine biosynthesis) expression, were related as a protective factor against muscular changes, such as myopathies, resulting from chronic use of statins [60]. Although the authors do not address the exact mechanism of action, it is expected that the decreased capacity for phosphocreatine storage in the muscle modifies the form of cellular energy storage, similar to that induced by glucose deprivation or potentially by cholesterol depletion [61,62].

Only PCO-C-treated Swiss mice showed significant upregulation in liver LCAT gene transcription, however without an increase in ApoA1 expression, which is an important activator of this enzyme. This result is relevant, since LCAT is a key enzyme in reverse cholesterol transport. LCAT efficiently esterifies free cholesterol, which is important in the process of maturation and remodeling of lipoproteins [60,63,64].

Interestingly, ApoE^−/−^ mice fed with HFD and with anthocyanin-rich extract showed an up-regulation of hepatic LCAT mRNA [65]. These results raise the hypothesis that the modulation of LCAT expression might be a mechanism of action that is common to different polyphenols. In addition, this finding is reinforced by the fact that PCO-C binds to LCAT, as shown by the docking molecular test, which suggests a positive feedback regulating LCAT function.

The docking test showed that PCO-C bound to both nLCAT (normal LCAT) and mLCAT (LCAT mutant). Many mutations have been identified in the LCAT enzyme [64,65]. Some of these mutations disrupt the geometric axis of the enzyme, thus incapacitating the complementarity of the enzyme with the substrates generating diseases, such as familial deficiency (FLD) and fish eye [66,67].

PCO-C was found with a low perturbation to the system and high affinity of the compound to the mLCAT active sites, with interaction energy ΔG: −8.78 Kcal/mol. The nLCAT enzyme with interaction energy ΔG: −7.04 Kcal/mol showed the participation of the residue Ser181 of the catalytic triad (Ser181, Asp345, His37) with the PCO-C; this amino acid acts as the nucleophil and is acylated during the stage of LCAT activation evidencing a possible mechanism of action [68].

Finally, our study reports the participation of the residues of the flexible lid (residues 226 to 246) that can change the accessibility of the catalytic site to its substrates [5,38] in the interaction with PCO-C, being three residues (Glu242; Ile245Thr246) of mLCAT and three residues (Asp227; Gln229 Ile245) nLCAT, highlighting the Asp227 aminoacid of the nLCAT that contributes to the specificity of the substrate [33].

One limitation of this study was to address in depth the mechanisms of action of this compound, its pharmacodynamics and pharmacokinetics, other possible therapeutic targets, and further effects on other genes that are involved in lipid metabolism, although a positive therapeutic effect of PCO-C against dyslipidemia was found. In addition, we have not addressed the potential effect of PCO-C to protect against atherosclerosis and other potential antioxidant properties.

## 5. Conclusions

In conclusion, infrared spectrometry and ^1^H-NMR further characterized the p-methoxycinnamic acid diester from carnauba wax powder (PCO-C) and subjected to toxicological tests. Our data suggest that PCO-C shows no cytotoxic effect against human peripheral blood lymphocytes (HPBLs). The alkaline comet assay and the micronucleus test showed no genotoxic activity in HPBLs and mice, both in vitro and in vivo.

In addition, PCO-C was effective in reducing serum TC and LDL-C levels, as well as promoting antioxidant protection of hepatic tissue without causing changes in hepatic and renal markers of toxicity. PCO-C also prevented excessive body weight- gain and food intake following a high-fat diet. PCO-C promoted the up-regulation of LCAT, which is an important enzyme for lipid mobilization through the reverse transport of cholesterol. Altogether, our findings suggest that the PCO-C might be an effective new compound in improving dyslipidemia, without detectable side effects. More studies are warranted to investigate other potential mechanisms that are involved in this protection.

## Figures and Tables

**Table 1:** Composition of the experimental standard chow diet.

Composition	Standard Chow Diet * (%)	HFD
Moisture	13.00	7.76
Crude protein	23.00	14.87
Ethereal extract	3.00	11.84
Fibrous matter	10.00	13.88
Carbohydrate	43.50	43.45
Mineral matter	7.50	8.20
Total	100	100

* MP 77 rodent diet from Primor^®^, Sao Paulo, Brazil.

**Table 2:** List of primers used for real-time PCR analysis.

Gene	Primer	Sequence (5′-3′)
ApoAI	Forward	TCAAAGACAGCGGCAGAGAC
ApoAI	Reverse	CACCTTCTGGCGGTAGAGCTC
LCAT	Forward	GTAACCACACACGGCCTGTCAT
LCAT	Reverse	GTTGAAATCCAGCCAGATGGT
LXRα	Forward	GCTCTGCTCATTGCCATCAG
LXRα	Reverse	TGTTGCAGCCTCTCTACTTGGA
HMGCR	Forward	CCGGCAACAACAAGATCTGTG
HMGCR	Reverse	ATGTACAGGATGGCGATGCA
HPRT	Forward	AGCTACTGTAATGATCAGTCAACG
HPRT	Reverse	AGAGGTCCTTTTCACCAGCA
B2M	Forward	CATGGCTCGCTCGGTGACC
B2M	Reverse	AATGTGAGGCGGGTGGAACTG

**Table 3:** Chemical shift, ^1^H-NMR spectral data of 4-methoxycinnamic acid diester (PCO-C) with the corresponding numbering.

Chemical Shift (ppm)	Assignments
7.62	8 (d, 1 H, 16 Hz)
7.45	5, 3 (m, 2 H)
6.85	6, 2 (m, 2 H)
6.33	9 (d, 1 H, 16 Hz)
3.83, 3.90	7 (s, 3 H)

X + Y = 58 mean value.

**Table 4:** Effects of different concentrations of PCO-C on the in vitro micronucleus tests after 24 h of treatment of human peripheral blood lymphocytes.

Treatment	MN ^a^
Negative Control	0.66 ± 0.33
Positive Control	25.66 ± 3.52 *
PCO-C 10 µg/mL	0.33 ± 0.03
PCO-C 50 µg/mL	0.66 ± 0.33
PCO-C 150 µg/mL	0.00 ± 0.00
PCO-C 250 µg/mL	0.33 ± 0.33

^a^ Number of micronuclei per 2000 binucleated cells analyzed. The negative control was treated with vehicle only (0.4% Tween-80); the positive control was treated with doxorubicin (0.3 µg/mL); * *p* < 0.05 compared to its respective negative control group (vehicle) according to the ANOVA followed by *Student-Newman-Keuls*.

**Table 5:** Effects of PCO-C and simvastatin treatment on serum levels lipids and glycaemia in experimental mice fed either a high fat diet (HFD) or standard diet (STD) for 30 and 90 days.

Serum Levels (mg/dL)	STD	HFD	SIMV	PCO-C
**Total Cholesterol**				
Baseline	99.29 ± 6.63	194.7 ± 0.97 ^a^	199.0 ± 5.08 ^a^	215.6 ± 11.88 ^a^
30 days	155.7 ± 3.73 *	243.1 ± 7.22 ^a,^*	201.4 ± 3.95 ^a,b^	197.3 ± 6.06 ^a,b^
90 days	144.1 ± 2.70 *	234.7 ± 16.18 ^a,^*	179.7 ± 8.36 ^a,b^	189.7 ± 4.82 ^a,b,^*
**Triglycerides**				
Baseline	104.6 ± 9.39	70.43 ± 5.19 ^a^	102.9 ± 4.46 ^b^	110.6 ± 5.40 ^b^
30 days	170.0 ± 20.35 *	121.3 ± 13.63 *	98.86 ± 6.29 ^a^	73.00 ± 7.02 ^a^
90 days	219.3 ± 26.11 *	194.1 ± 28.51 *	124.7 ± 15.74 ^a^	153.6 ± 17.87
**HDL-C**				
Baseline	80.00 ± 2.25	78.29 ± 2.08	86.71 ± 2.12	78.43 ± 2.94
30 days	87.71 ± 2.00	115.30 ± 7.53 ^a,^*	105.70 ± 6.93	78.57 ± 4.74 ^b^
90 days	59.29 ± 13.31	42.43 ± 14.00 *	84.71 ± 6.40 ^b^	87.57 ± 4.02 ^b,c^
**LDL-C**				
Baseline	31.00 ± 3.96	102.30 ± 2.34 ^a^	91.71 ± 5.61 ^a^	115.00 ± 12.46 ^a^
30 days	34.00 ± 4.02	103.60 ± 7.57 ^a^	75.94 ± 5.50 ^a,b^	104.10 ± 6.25 ^a^
90 days	41.00 ± 10.20	153.50 ± 22.28 ^a,^*	70.06 ± 1.82 ^a,b^	83.86 ± 11.84 ^a,b,c^
**Glycemia**				
Baseline	105.60 ± 7.72	102.40 ± 4.17	101.10 ± 4.01	99.43 ± 3.90
30 days	91.43 ± 5.86	93.86 ± 6.15	83.29 ± 3.26	96.43 ± 3.85
90 days	115.90 ± 7.35	108.40 ± 9.62	101.40 ± 4.09	120.1 ± 6.35 *

Baseline = immediately post-induction of dyslipidemia; STD = standard diet; HFD = high fat diet; SIMV = simvastatin (20 mg/Kg/day). PCO-C values are given the mean ± SEM of 7 mice per group. ^a^ = *p* < 0.05 versus the STD group; ^b^ = *p* < 0.05 versus HFD group; ^c^ = *p* < 0.05 versus SIMV group; * *p* < 0.05 versus intra-group baseline.

**Table 6:** Effects of PCO-C on weight gain and food intake of mice that were subjected to the hypercholesterolemic diet and controls.

Groups	Initial Weight (g)	Final Weight (g)	Total Weight Gain (g)	Food Intake (g/Mice/Day)		
				30 days	60 days	90 days
STD	32.09 ± 1.02	45.87 ± 1.22	13.79 ± 0.77	5.29 ± 0.12	5.52 ± 0.17	5.34 ± 0.07
HFD	32.47 ± 0.90	51.47 ± 1.44 ^a^	19.00 ± 1.94 ^a^	5.22 ± 0.22	5.38 ± 0.25	5.46 ± 0,07
SIMV	31.33 ± 0.85	47.46 ± 1.14 ^b^	16.13 ± 0.80	4.98 ± 0.20	5.08 ± 0.18	5.09 ± 0.08 ^b^
PCO-C	31.70 ± 0.65	42.87 ± 0.79 ^b^	12.25 ± 0.55 ^b^	5.03 ± 0.05	4.85 ± 0.15	4.98 ± 0.04 ^a,b^

STD = standard chow diet; HFD = high fat diet; SIMV = simvastatin (20 mg/Kg/day); PCO-C = *p*-methoxycinnamic acid diesters (100 mg/Kg/day). Values are given as mean ± SEM of 7 mice per group. ^a^ = *p* < 0.05 versus the STD group; ^b^ = *p* < 0.05 versus HFD group.

**Table 7:** Effects of PCO-C on the relative liver weight, malondialdehyde and kidney and hepatic toxicity markers in mice fed with HFD and controls.

Parameters	STD	HFD	SIMV ^‡^	PCO-C
RLW (mg)	3.47 ± 0.14	4.67 ± 0.25 ^a^	4.14 ± 0.17 ^b^	4.20 ± 0.10 ^b^
MDA (nmol/g)	0.24 ± 0.02	0.39 ± 0.05 ^a^	^_^	0.26 ± 0.03 ^b^
AST (U/L)	77.00 ± 9.51	48.43 ± 1.07	211.1 ± 11.05 ^a,b^	48.14 ± 4.15
ALT (U/L)	35.43 ± 3.49	35.71 ± 2.90	47.14 ± 3.64	44.29 ± 4.39
UREA (mg/dL)	48.71 ± 2.00	55.86 ± 1.22	52.71 ± 1.81	49.86 ± 2.55
CREAT	0.81 ± 0.06	0.70 ± 0.07	0.49 ± 0.04 ^a^	0.60 ± 0.05

STD = standard chow diet; HFD = high fat diet; SIMV = simvastatin (20 mg/Kg/day); PCO-C = *p*-methoxycinnamic acid diesters (100 mg/Kg/day). RLW = relative liver weight (RLW = liver weight in percent of body weight); MDA = malondialdehyde; AST = aspartate aminotransferase; ALT = alanine aminotransferase; CREAT = creatinine. Values are given as mean ± SEM of 7 mice per group. ^a^ = *p* < 0.05 versus the STD group; ^b^ = *p* < 0.05 versus HFD group. ^‡^ Missing data for MDA analyses.

**Table 8:** Interaction energy of PCO-C and dithiobis-nitrobenzoic acid (DTNB) with Lecithin Cholesterol Acyltransferase (LCAT) with and without the mutation (Cys31-Tyr31).

	Protein (mLCAT)		Protein (nLCAT)	
	ΔG: Kcal/mol	Ki	ΔG: Kcal/mol	Ki
PCO-C	−8.78	365.47 nM	−7.04	6.88 uM
DTNB	−11.44	4.13 nM	−10.03	44.64 nM

Ki = inhibition coefficient.
